# Deep Learning Approach in Image Diagnosis of *Pseudomonas* Keratitis

**DOI:** 10.3390/diagnostics12122948

**Published:** 2022-11-25

**Authors:** Ming-Tse Kuo, Benny Wei-Yun Hsu, Yi Sheng Lin, Po-Chiung Fang, Hun-Ju Yu, Yu-Ting Hsiao, Vincent S. Tseng

**Affiliations:** 1 Department of Ophthalmology, Kaohsiung Chang Gung Memorial Hospital and Chang Gung University College of Medicine, Kaohsiung 83301, Taiwan; 2 School of Medicine, Chang Gung University, Taoyuan 33302, Taiwan; 3 Department of Computer Science, National Yang Ming Chiao Tung University, Hsinchu 300, Taiwan

**Keywords:** image diagnosis, *Pseudomonas* keratitis, bacterial keratitis, microbial keratitis, deep learning, ensemble learning, machine learning, artificial intelligence

## Abstract

This investigation aimed to explore deep learning (DL) models’ potential for diagnosing *Pseudomonas* keratitis using external eye images. In the retrospective research, the images of bacterial keratitis (BK, *n* = 929), classified as *Pseudomonas* (*n* = 618) and non-*Pseudomonas* (*n* = 311) keratitis, were collected. Eight DL algorithms, including ResNet50, DenseNet121, ResNeXt50, SE-ResNet50, and EfficientNets B0 to B3, were adopted as backbone models to train and obtain the best ensemble 2-, 3-, 4-, and 5-DL models. Five-fold cross-validation was used to determine the ability of single and ensemble models to diagnose *Pseudomonas* keratitis. The EfficientNet B2 model had the highest accuracy (71.2%) of the eight single-DL models, while the best ensemble 4-DL model showed the highest accuracy (72.1%) among the ensemble models. However, no statistical difference was shown in the area under the receiver operating characteristic curve and diagnostic accuracy among these single-DL models and among the four best ensemble models. As a proof of concept, the DL approach, via external eye photos, could assist in identifying *Pseudomonas* keratitis from BK patients. All the best ensemble models can enhance the performance of constituent DL models in diagnosing *Pseudomonas* keratitis, but the enhancement effect appears to be limited.

## 1. Introduction

Microbial keratitis (MK) is a vision-threatening corneal infectious disease. MK is generally divided into viral, bacterial, fungal, and parasitic keratitis [1]. Among the different MKs, bacterial keratitis (BK) is an emergent disease, which is more painful and rapidly progressive than other MKs [2,3]. *Pseudomonas*, one of the most common pathogens in BK, quickly infects the cornea through contact lens wear or contaminated water exposure [4,5]. Compared to other BKs, *Pseudomonas* keratitis is more fulminant in its clinical progression [6]. A late diagnosis of BK, especially in *Pseudomonas* keratitis, may lead to corneal melt and subsequent perforation. Hence, quick diagnosis of *Pseudomonas* keratitis from a suspected BK patient is highly critical.

Diagnosing MKs via an image-based convolutional neural network (CNN) model holds promise in frontline healthcare facilities with no available ophthalmologists [7,8,9]. Thus, we developed and compared eight up-to-the-minute CNN models through deep learning (DL) to differentiate BK from other MKs [10]. We found no statistical difference in area under the receiver operating characteristic curve (AUROC; range from 73% to 77%) and diagnostic accuracy (range from 69% to 72%) among these algorithms. However, during that preliminary work, we had not yet explored the potential of these CNN algorithms to differentiate *Pseudomonas* from non-*Pseudomonas* BK. In the literature review, no artificial intelligence (AI) models were developed to diagnose *Pseudomonas* keratitis.

Ensemble approaches, which use multiple learning algorithms to perform the same classification task, could yield better validity and an improved generalization performance compared to constituent learning algorithms when there is an apparent diversity among the models [11]. The CNN-based ensemble approach could take advantage of different CNN algorithms, which have a variety of characteristics in the learning process, instead of conventional ensemble approaches that use similar settings and lots of weak learners (e.g., machine learning models) to build the final model. It was reported that the ensemble DL model could better classify the stages of diabetic retinopathy and glaucoma using fundus photos than a solo CNN model [12,13].

No AI model has been established to identify *Pseudomonas* keratitis, which carries a high risk of degrading vision. At least as a proof of concept, we hope to fill this gap by identifying *Pseudomonas* keratitis from bacteria keratitis via imaging DL models. Given the potential of state-of-the-art DL algorithms and the advantage of the ensemble DL model, this study aimed to explore single and ensemble DL models’ imaging capabilities whe n diagnosing *Pseudomonas* keratitis.

## 2. Materials and Methods

**Figure 1:**
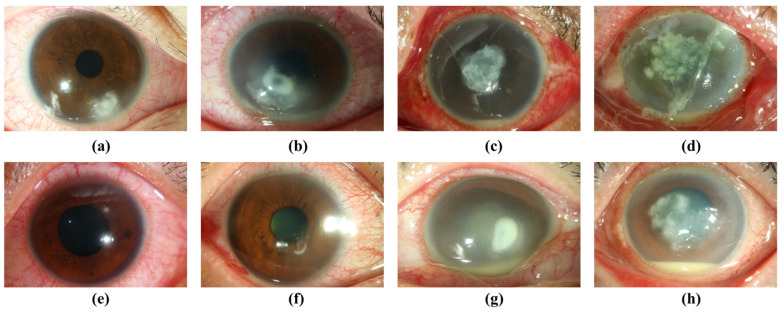
Representative photographs of bacterial keratitis caused by *Pseudomonas* and non-*Pseudomonas* spp. (**a**–**d**) *Pseudomonas* keratitis; (**e**) *Serratia* keratitis; (**f**) *Staphylococcus* keratitis; (**g**) *Streptococcus* keratitis; (**h**) non-tuberculosis *Mycobacterium* keratitis.

A total of 929 external eye images from 929 BK patients photographed by camera-mounted slit lamp biomicroscope were obtained from Chang Gung Research Database, retrospectively collecting data from 5 major Chang Gung Memorial Hospital branches in Taiwan [10]. Each photo was collected from an individual patient. These images were photographed under white light illumining without slit-lamp enhancement (Figure 1). The research obeyed the ARVO statement on human subjects and the Declaration of Helsinki. It was permitted by the Institutional Review Board of Chang Gung Medical Foundation (approval code: 201901255B0C601), which waived the informed consent for subjects in the investigation due to our protection of privacy by delinking personal identification at data and image analysis and the essence of a retrospective research.

The subjects enrolled for BK must fulfill one of the following criteria: First, at least one of the following laboratory tests confirmed with a bacterial pathogen, including dye-staining light microscopy (acid-fast or Gram stain), microbial culture (chocolate agar, blood agar, Löwenstein–Jensen slant, or Sabouraud dextrose agar) and DNA tests (dot hybridization assay, or polymerase chain reaction) for corneal scrapes, and histopathological examination for the sample of corneal biopsy [4,14]. Second, 3 corneal experts (≥8 years of specialty) made a concordant impression of *Pseudomonas* or non-*Pseudomonas* keratitis for the same BK case. The subject was excluded if: contaminated microorganisms such as *Micrococcus* spp. or *Staphylococcus epidermidis*, or mixed infections were reported by laboratory tests; 3 corneal experts could not reach a concordant impression. Accordingly, this experiment classified these photos of BK into *Pseudomonas* keratitis (*n* = 618) and non-*Pseudomonas* keratitis (*n* = 311).

The image-preprocessing procedure was the same as in our previous study [7]. Briefly, the identification information and date of photographing in the photos were automatically pre-cut with specially designed software via batch-processing. All input photos were adjusted to 224 × 224 pixels’ size. We normalized each pixel’s RGB values of an image from 0 to 1 before going into the CNN training process.

**Figure 2:**
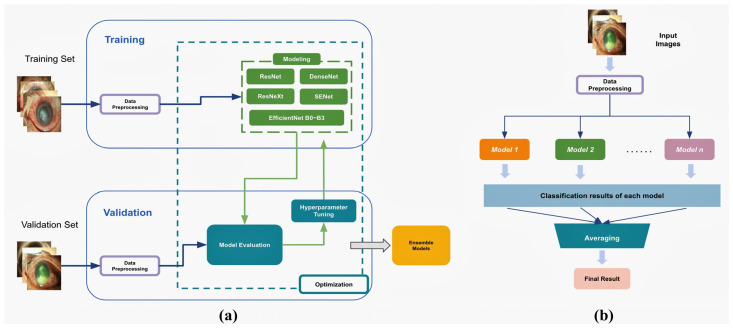
The framework of deep learning models for identifying *Pseudomonas* keratitis by external eye photographs. (**a**) The input images were split into training and validation sets. ResNet50, ResNeXt50, DenseNet121, SE-ResNet50, and EfficientNet B0, B1, B2, and B3 were adopted as the constituent backbone models. After data preprocessing, we optimize the framework through training CNN-based backbone models, evaluating the validation set, and hyperparameter tuning. The optimized models were used for the further ensemble process. (**b**) The ensemble model combines *n* predicted probabilities from various models and calculates the average probability. Here, “*n*” represents the number of models selected in the ensemble combinations.

All 929 BK images were used as the dataset to establish diagnostic models of *Pseudomonas* keratitis. The single model used for detecting *Pseudomonas* keratitis was constructed similarly to our previous study’s model for diagnosing BK [10]. Figure 2a illustrates the entire pipeline of our framework. We used the training images to train a DL model to distinguish *Pseudomonas* from non-*Pseudomonas* keratitis. In contrast, we used the validation images to test the capability of a trained model. In modeling, we used various CNN-based methods to produce base models for further ensemble. To create the ideal model, the hyperparameters of each model were tuned empirically, such as batch size and learning rate, according to the validation results. Figure 2b shows the detail of the ensemble model for diagnosing *Pseudomonas* keratitis. We combined *n* predicted probabilities and calculated the average likelihood as a result. At the validation stage, we remained with the top-performance DL models as candidates and selected *n* models to arrange the ensemble model. After testing all combinations (*n* = 2, 3, 4, 5), we obtained the best ensemble 2-, 3-, 4-, and 5-DL (BE2, BE3, BE4, and BE5) models. Ensemble diagnostic models were trained toward the target with AUROC. We established the models in PyTorch 1.4.0 and implemented all the above experiments on GeForce RTX 1080 GPUs of NVIDIA (Santa Clara, CA, USA).

We used k-fold cross-validation (k = 5) to determine the performance of DL diagnostic models. The accuracy, positive predictive value (PPV), sensitivity, negative predictive value (NPV), and specificity were used as the indexes of diagnostic validity. The photos per group were equally and randomly allocated into 5 datasets. There were 123–124 images of *Pseudomonas* keratitis and 62–63 pictures of non-*Pseudomonas* keratitis in respective datasets. Four of the 5 datasets were used for training a CNN model, and the residual one was used for validating this model.

The average validity indexes and AUROC of diagnosing *Pseudomonas* keratitis were compared for the single and the best ensemble DL models. Fisher’s exact test compared the validity index between any two models. Furthermore, the Z score test determined the statistical significance of AUROC between two different models. *p* < 0.05 was considered a significant difference. GraphPad Prism version 9.4.1 (GraphPad Software, San Diego, CA, USA) was used to perform the above statistical task.

## 3. Results

### 3.1. The Performance of a Single DL Model in Diagnosing Pseudomonas Keratitis

**Figure 3:**
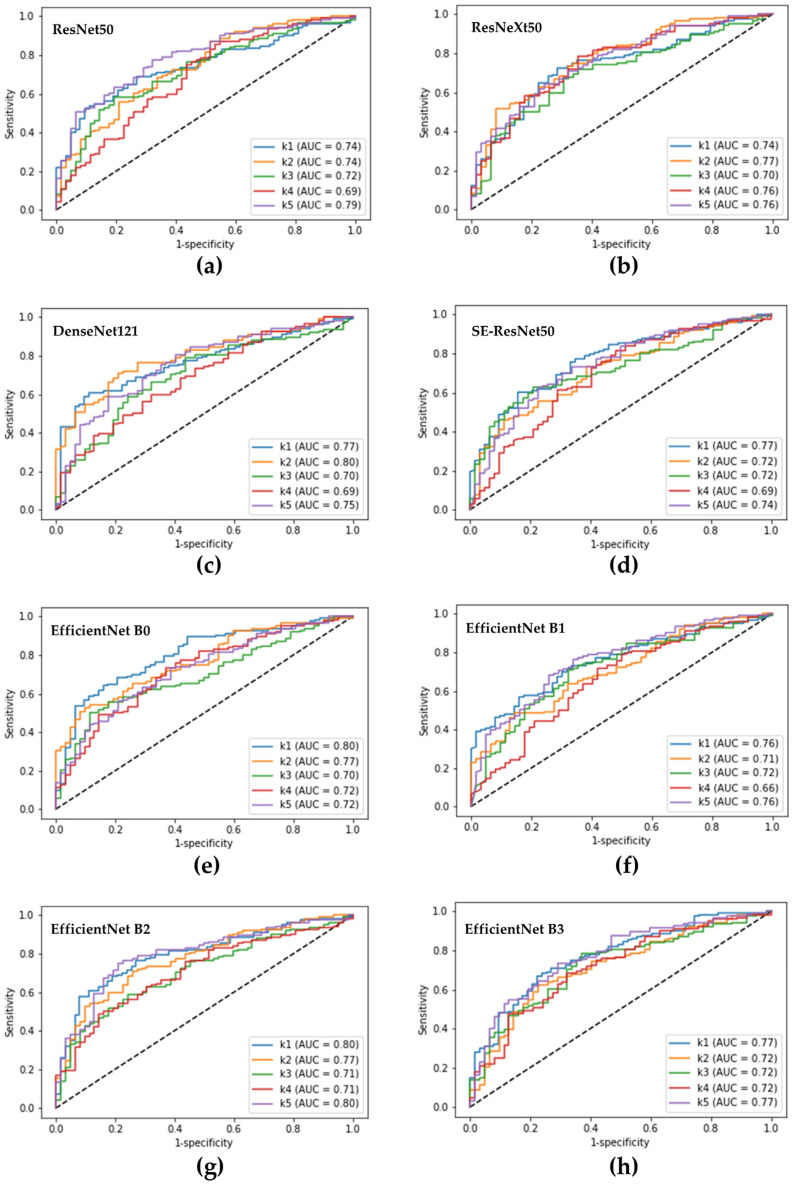
Receiver operating characteristic curves obtained from the fivefold cross-validation of the single deep learning models for distinguishing pseudomonas keratitis from other forms of bacterial keratitis. (**a**) Receiver operating characteristic (ROC) curves of the ResNet50 model; (**b**) ROC curves of the ResNeXt50 model; (**c**) ROC curves of the DenseNet121 model; (**d**) ROC curves of the SE-ResNet50 model; (**e**–**h**) ROC curves of the EfficientNets B0, B1, B2, B3 models, respectively. k1 to k5 represent the obtained ROC curves from the five test results of fivefold cross-validation. AUC, the area under the ROC curve.

The ability of a single DL model to diagnose *Pseudomonas* keratitis is revealed in Table 1. For the 8 DL models, the EfficientNet B2 model showed the highest accuracy (71.2%) with 81.1% sensitivity, 51.5% specificity, 76.9% PPV, and 57.9% NPV. The DenseNet121 model had the most heightened sensitivity (82.5%), and the EfficientNet B3 model had the highest PPV (81.7%) and specificity (68.2%). The non-EfficientNet models performed similarly to the EfficientNet B2 model, with significantly higher sensitivities than EfficientNet models B0, B1, and B3. In contrast, EfficientNets B0 and B3 models demonstrated statistically higher specificities than the EfficientNet B2 and non-EfficientNet models. Moreover, the EfficientNet B3 model had a significantly higher PPV than the four non-EfficientNet models. However, no significant difference was discovered among the eight models in NPV, accuracy, and AUROC (Figure 3).

### 3.2. The Performance of an Ensemble Model for Recognizing Pseudomonas Keratitis

**Figure 4:**
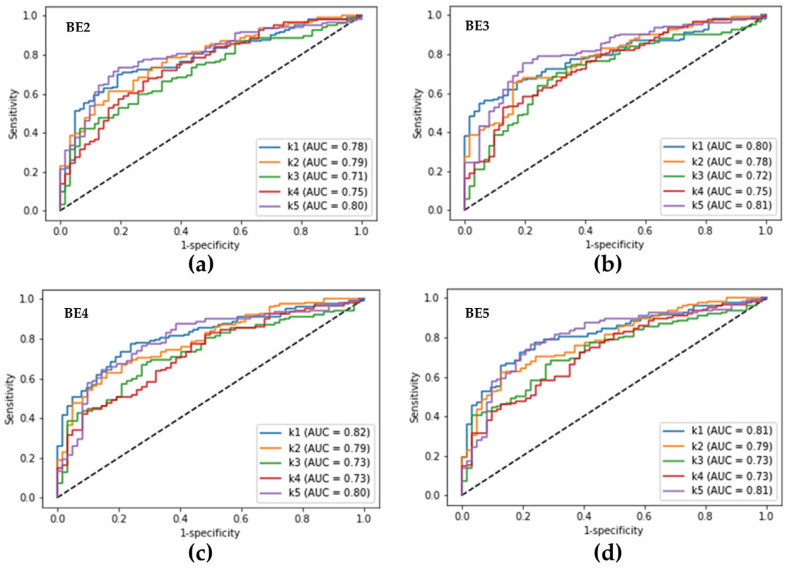
Receiver operating characteristic curves obtained from the fivefold cross-validation of the best ensemble models for diagnosing Pseudomonas keratitis. (**a**) Receiver operating characteristic (ROC) curves of the best ensemble 2-deep learning model; (**b**) ROC curves of the best ensemble 3-deep learning model; (**c**) ROC curves of the best ensemble 4-deep learning model; (**d**) ROC curves of the best ensemble 5-deep learning model. BE2, BE3, BE4, and BE5 represent the best ensemble 2-, 3-, 4-, and 5-deep learning models. k1 to k5 represent the obtained ROC curves from the five test results of fivefold cross-validation. AUC, the area under the ROC curve.

The average diagnostic performance of the best ensemble model for identifying *Pseudomonas* keratitis in BK is shown in Table 2. The BE4 model had the highest accuracy (72.1%) among the four ensemble models, with 79.6% sensitivity, 57.2% specificity, 78.7% PPV, and 59.2% NPV. The BE3 model revealed the highest sensitivity (83.7%) and NPV (60.1%), whereas the BE5 model had the highest specificity (57.9%) and PPV (78.9%). Furthermore, the BE3 model showed a significantly higher sensitivity than the BE5 model, whereas models BE4 and BE5 had higher specificities than models BE2 and BE3. However, there was no significant difference among the four ensemble models in PPV, NPV, accuracy, and AUROC (Figure 4) in diagnosing *Pseudomonas* keratitis.

### 3.3. Comparing Ensemble with Single DL Models in Identifying Pseudomonas Keratitis in BK

**Figure 5:**
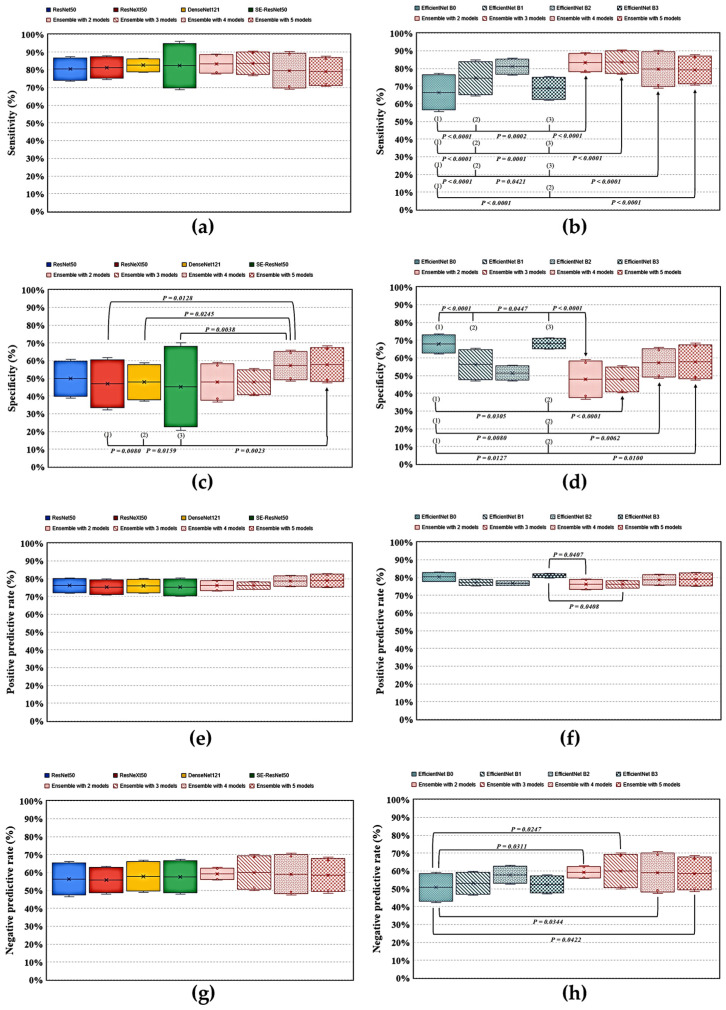
Comparison of the performance of ensemble models with constituent deep learning models via an external eye photo for differentiating *Pseudomonas* keratitis from other bacterial keratitis. (**a**,**b**) Diagnostic sensitivity of different models; (**c**,**d**) diagnostic specificity of the models; (**e**,**f**) positive predictive value of the models; (**g**,**h**) negative predictive value of the models. The arrow indicates a comparison between the pointed model and the model with a parenthesized number. *p* < 0.05 was recognized as a statistical difference.

**Figure 6:**
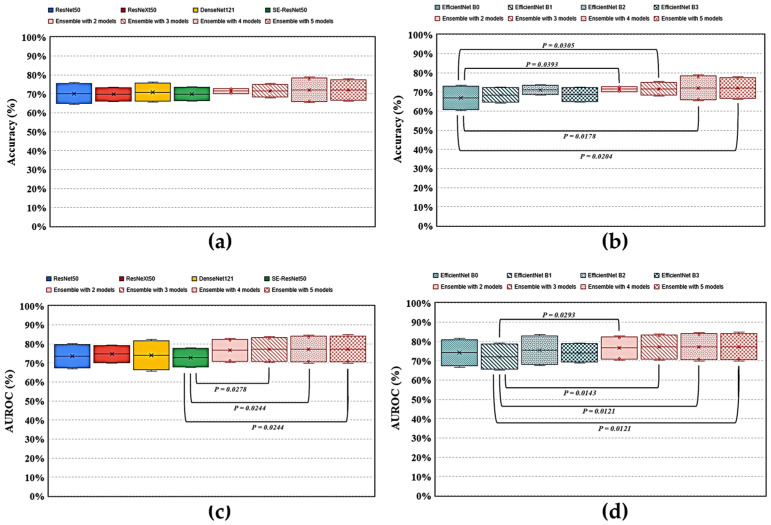
Comparison of the performance of ensemble models with constituent deep learning models via an external eye photo for differentiating *Pseudomonas* from non-*Pseudomonas* keratitis. (**a**,**b**) Diagnostic accuracy of different models; (**c**,**d**) area under the receiver operating characteristic curve (AUROC) of the models. *p* < 0.05 was recognized as a statistical difference.

When comparing the ensemble models BE4, which had the highest accuracy among the four best ensemble models in distinguishing *Pseudomonas* keratitis from other BKs (Table 2), with the eight backbone DL models (Table 1), the BE4 model had a significantly higher sensitivity than EfficientNets B0, B1, and B3 (Figure 5). In addition, this model had a significantly higher specificity than ResNeXt50, DenseNet121, and SE-ResNet50, while it showed a significantly lower specificity than EfficientNets B0 and B3. Moreover, the BE4 model revealed a significantly higher NPV than ResNet50. Furthermore, this ensemble model had a higher accuracy than EfficientNet B0 and a higher AUROC than SE-ResNet50 and EfficientNet B1 in statistics (Figure 6).

## 4. Discussion

In Taiwan and many other areas globally, BK is the most common MK and the main cause of corneal opacification leading to vision loss, in which *Pseudomonas* is the major pathogen with high corneal virulence [1,14,15,16]. Moreover, *Pseudomonas* is the most common pathogen of MK in Asia-Pacific and the population of contact-lens wearers [5,16]. The rapid identification of *Pseudomonas* keratitis from suspected BK is critical to prevent corneal ulcer patients from corneal melting and blindness. Diagnosing BK from MK via the DL approach of external eye photos has recently shown promise [8,9,10,17]. Still, there is no report exploring the potential of DL models for further differentiating BK into *Pseudomonas* and non-*Pseudomonas* keratitis to date. In this study, we adopted eight state-of-art DL algorithms and their ensemble DL models to elucidate the DL technology’s ability to identify *Pseudomonas* keratitis from suspected BK. We found that ensemble models can promote the diagnostic ability of single DL models to distinguish *Pseudomonas* keratitis from other BKs. The ensemble models in differentiating *Pseudomonas* with non-*Pseudomonas* BK also showed higher accuracy (71.5–72.1%) than those (67.0–71.2%) of the constitutive models. Moreover, these models had a higher AUROC (76.6–77.3%) than those (72.2–75.6%) of their backbone models.

This study is the first to develop a CNN model to differentiate *Pseudomonas* keratitis from non-*Pseudomonas* BK. Ophthalmologists’ sensitivity and PPV, based on the clinical diagnosis of *Pseudomonas* keratitis were 75% and 65%, respectively [18]. We found the performance of DL models developed in this study is not inferior to ophthalmologists. Some single DL models (ResNet50, ResNeXt50, DenseNet121, SE-ResNet50, and EfficientNet B2) revealed comparable sensitivity (80.4–82.5% vs. 79.1–83.3%) and PPV (75.2–76.9% vs. 76.1–78.9%) to the four best ensemble models (Table 1 and Table 2, and Figure 5 and Figure 6). However, we should notice that the image database was the same as in our previous study [10]; it had an inherent limitation in clinical diagnosis consensus, established by three experienced corneal specialists’ reviews on patients’ clinical presentations and treatment histories. Few subjects may be unavoidably misclassified.

Recently, Ghosh et al. described an ensemble CNN architecture, composed of VGG19, ResNet50, and DenseNet121, to differentiate BK from fungal keratitis [9]. This ensemble model can reach a sensitivity and PPV of 77% and 91%. For the same purpose, Redd et al. reported that an ensemble CNN model consisting of Xception, VGG, ResNet, DenseNet, and MobilNet could reach accuracy and AUROC of 75% and 84% in diagnosing BK [17]. The two studies presented that the ensemble DL model outperforms the composed CNN models in diagnosing BK. We employed any combinations from eight state-of-the-art CNN algorithms to clarify the limits of an ensemble DL model in recognizing *Pseudomonas* keratitis from BK (Table 2 and Figure 4). The four best ensemble models revealed the highest sensitivity (84%) in the BE3 model and the highest PPV (79%) in the BE5 model, while the lowest sensitivity (76%) was revealed in the BE5 model and the lowest PPV (76%) in the BE2 model (Table 2). Moreover, the four ensemble models demonstrated a similar diagnostic accuracy and AUROC, whereas the BE4 model showed the highest accuracy (72.1%) and AUROC (77.3%) (Table 2 and Figure 4). This implies that distinguishing *Pseudomonas* keratitis from non-*Pseudomonas* BK is more difficult than differentiating BK from FK.

Some studies described other ways to promote the CNN-based diagnostic performance based on imaging in BK [8,19,20]. A deep sequential feature learning approach was adopted by Xu et al. to identify BK from MK. Their model reached up to 80% diagnostic accuracy and 94% AUROC, with a manual annotation and patch sampling procedure of images before DL [19]. Hung et al. used two classes of external eye images (direct illumination and fluorescence-enhancing) and an extra U2 Net model for segmentation before feature extraction and classification of BK and fungal keratitis with the DenseNet161 model [8]. The overall diagnostic accuracy and AUROC attained 79% and 85%, with a sensitivity of 66% and a PPV of 74%. Koyama et al. also used two types of external eye images. They developed a hybrid learning approach consisting of a ResNet50 and InceptionResNetV2 architectures’ hierarchy and a gradient boosting decision tree, a machine learning algorithm [20]. They reported that this hybrid learning model had an accuracy of 91% and an AUROC of 96%, which was an accurate method for the computer-assisted diagnosis of MK. We believe the above techniques could help to enhance a DL model for diagnosing *Pseudomonas* keratitis from suspected BK.

We used the eight DL algorithms because we had tested the feasibility of these algorithms on ocular images in our preliminary study [10]. We used ResNet due to the simple concept of residual blocks that make the CNN architecture deeper, without degradation problems. ResNet is also a baseline method in most DL-related studies. Therefore, DenseNet and ResNeXt, considered an enhanced versions of ResNet and used in many applications, should be included to test the performance. SENet is the representative method that first uses a channel-attention mechanism in other CNNs (e.g., ResNet and ResNeXt) to boost performance. EfficientNets was proposed based on the neural architecture search (NAS) method and generated by refining the dimensions of CNNs (e.g., depth, width, and resolution).

It is essential to note that a DL model learning from the impressions of experts may reduce interference from uncharacteristic manifestations of several BK patients. Nevertheless, many prior DL models accepted experts’ impressions as a gold standard [21,22,23]. Moreover, comparing the performance between ensemble models and their constituent DL models is objective and fair when using the same image database. Therefore, we adopted the database in this study.

In addition, ensemble methods are always composed of different learners, making it hard to track the contribution of every learner. Thus, conducting a series of experiments is necessary to validate and acquire the best combination. The powerful ensemble approach generated remarkable classification results. Nevertheless, constructing a solid ensemble model requires many computation resources, including time and storage capacity. We had to train several DL models, analyze different combinations of the models in advance, and then produce the final, robust model. Hence, the ensemble model is appropriate for applications of cloud-based, computer-aided diagnosis and areas limited to portable applications, which is another promising applied scenario that we may consider developing in the future.

This study demonstrated the feasibility of concept that the application of advanced DL models to differentiate *Pseudomonas* from non-*Pseudomonas* BK is promising. This model could be useful in rapid image diagnosis and treatment for patients infected with fulminant *Pseudomonas* keratitis in clinical settings without ophthalmologists in the future. However, we just enrolled subjects with BK. Therefore, the result of this study cannot infer the same performance of DL models in differentiating *Pseudomonas* keratitis from non-bacterial MK. Moreover, we did not adopt external image sets to demonstrate the models’ generality, which is critical to prove their clinical feasibility. Nonetheless, the training and validation used images from five hospitals with different clinical settings. The five-fold cross-validation design could accurately estimate the average performance of each model and is suitable for a proof of concept.

## 5. Conclusions

Based on imaging, both single and ensemble DL approaches could provide an alternative way to identify *Pseudomonas* keratitis from suspected BK. Without requiring additional fluorescence dye-staining photos, manual annotation, proceeding segmentation of images, complicated computation, or hybrid techniques of deep and machine learning, the ensemble DL model offers an efficient means of distinguishing *Pseudomonas* keratitis from other BKs based only on external eye images and constituent DL models. As a proof of concept, this approach could be more intuitive and valuable in promoting the diagnostic limits of single DL models in diagnosing *Pseudomonas* keratitis. There was no previous study addressing the image diagnosis of *Pseudomonas* keratitis using a CNN model. The main contribution of the study to the literature is to fill the gap by approving several advanced CNN models and their ensembles via ocular imaging to differentiate *Pseudomonas* from non-*Pseudomonas* BK.

## Figures and Tables

**Table 1:** Diagnostic performance of eight state-of-art deep learning models for identifying *Pseudomonas* keratitis in bacterial keratitis.

Model	Diagnostic Performance (95% Confidence Interval)				
	Sensitivity	Specificity	PPV	NPV	Accuracy
ResNet50	80.4	49.9	76.1	56.4	70.2
	(73.5~87.3)	(39.0~60.7)	(71.8~80.5)	(46.6~66.1)	(64.4~75.9)
ResNext50	81.2	46.9	75.4	55.8	69.8
	(74.6~87.9)	(32.2~61.7)	(70.8~79.9)	(48.1~63.5)	(65.8~73.7)
DenseNet121	82.5	47.9	75.9	57.9	70.9
	(78.5~86.6)	(37.0~58.8)	(71.7~80.1)	(49.0~66.9)	(65.7~76.1)
SE-ResNet50	82.4	45.3	75.2	57.6	70.0
	(68.7~96.1)	(20.54~70.1)	(70.0~80.5)	(47.9~67.4)	(66.2~73.7)
EfficientNet B0	66.5	67.9	80.4	50.8	67.0
	(55.7~77.4)	(62.2~73.5)	(77.6~83.2)	(42.4~59.2)	(60.3~73.6)
EfficientNet B1	74.6	56.3	77.3	53.2	68.5
	(64.4~84.8)	(47.0~65.6)	(75.3~79.2)	(46.4~59.9)	(64.3~72.6)
EfficientNet B2	81.1	51.5	76.9	57.9	71.2
	(76.3~85.8)	(47.1~55.8)	(75.4~78.3)	(52.6~63.2)	(68.5~73.8)
EfficientNet B3	68.8	68.2	81.1	52.5	68.6
	(62.0~75.5)	(65.0~71.3)	(79.8~82.4)	(47.2~57.8)	(64.6~72.5)

PPV = positive predictive value; NPV = negative predictive value.

**Table 2:** Diagnostic performance of ensemble deep learning models for identifying *Pseudomonas* keratitis in bacterial keratitis.

Model	Diagnostic Performance (95% Confidence Interval)				
	Sensitivity	Specificity	PPV	NPV	Accuracy
BE2	83.3	47.9	76.1	59.3	71.5
	(77.7–89.0)	(36.8–59.1)	(73.2–79.1)	(55.7–62.9)	(70.0–72.9)
BE3	83.7	47.9	76.2	60.1	71.7
	(76.7–90.6)	(40.3–55.5)	(73.9–78.4)	(50.0–70.2)	(68.0–75.4)
BE4	79.6	57.2	78.7	59.2	72.1
	(69.0–90.3)	(48.6–65.9)	(75.4–82.0)	(47.4–70.9)	(65.4–78.9)
BE5	79.1	57.9	78.9	58.6	72.0
	(70.6–87.7)	(47.5–68.3)	(74.9–82.9)	(48.5–68.6)	(66.1–78.0)

BE2, BE3, BE4, and BE5 represent the best ensemble model integrating two, three, four, and five deep learning models, respectively. PPV = positive predictive value; NPV = negative predictive value.

## Data Availability

The datasets generated and analyzed during the current study are available from the corresponding author on reasonable request.
